# Emergence of *Burkholderia pseudomallei* Sequence Type 562, Northern Australia

**DOI:** 10.3201/eid2704.202716

**Published:** 2021-04

**Authors:** Ella M. Meumann, Mirjam Kaestli, Mark Mayo, Linda Ward, Audrey Rachlin, Jessica R. Webb, Mariana Kleinecke, Erin P. Price, Bart J. Currie

**Affiliations:** Menzies School of Health Research, Darwin, Northern Territory, Australia (E.M. Meumann, M. Kaestli, M. Mayo, L. Ward, A. Rachlin, J.R. Webb, M. Kleinecke, E.P. Price, B.J. Currie);; Charles Darwin University, Darwin (E.M. Meumann, M. Kaestli, M. Mayo, L. Ward, A. Rachlin, J.R. Webb, M. Kleinecke, E.P. Price, B.J. Currie);; Royal Darwin Hospital, Darwin (E.M. Meumann, B.J. Currie);; University of the Sunshine Coast, Sippy Downs, Queensland, Australia (E.P. Price)

**Keywords:** melioidosis, genomics, epidemiology, *Burkholderia pseudomallei*, sequence type, Australia, phylogenetics, bacteria, ST562

## Abstract

Since 2005, the range of *Burkholderia pseudomallei* sequence type 562 (ST562) has expanded in northern Australia. During 2005–2019, ST562 caused melioidosis in 61 humans and 3 animals. Cases initially occurred in suburbs surrounding a creek before spreading across urban Darwin, Australia and a nearby island community. In urban Darwin, ST562 caused 12% (53/440) of melioidosis cases, a proportion that increased during the study period. We analyzed 2 clusters of cases with epidemiologic links and used genomic analysis to identify previously unassociated cases. We found that ST562 isolates from Hainan Province, China, and Pingtung County, Taiwan, were distantly related to ST562 strains from Australia. Temporal genomic analysis suggested a single ST562 introduction into the Darwin region in ≈1988. The origin and transmission mode of ST562 into Australia remain uncertain.

The tropical disease melioidosis causes sepsis in persons with risk factors such as diabetes or hazardous alcohol consumption (*1*). The causative bacterium, *Burkholderia pseudomallei*, is found in soil and surface waters. Most reported cases of melioidosis occur in Southeast Asia and northern Australia during the monsoonal wet seasons (i.e., November–April in northern Australia and May–October in Southeast Asia) (*1*). Although melioidosis is increasingly found in China, the Pacific Islands, South Asia, Africa, and Central and South America (*2*), laboratory diagnostic constraints contribute to underreporting of cases. As a result, the true global distribution and prevalence of melioidosis remain uncertain (*3*). 

Clinical manifestations are varied; however, pneumonia is the most common form, accounting for ≈50% of cases (*4*). The frequencies of these manifestations differ by region. For example, suppurative parotitis is common in children in Thailand and Cambodia but rare in Australia; manifestations such as prostate abscesses and brainstem encephalitis are reported rarely outside Australia (*1*,*4*). Death rates range from 9% in northern Australia to 40% in northeast Thailand (*1*,*4*). The extent to which transmission mode, host risk factors, access to diagnostic testing, appropriate antimicrobial drugs, and intensive care treatment account for differences in manifestations and outcomes remains uncertain. Clinical studies suggest that host risk factors are major contributors to disease severity and outcome (*1*).

Phylogeographic analyses suggest that *B. pseudomallei* emerged in ancient Australia and subsequently disseminated throughout Asia (*2*,*5*,*6*). Because of their ecologic niche, sensitivity to ultraviolet light, and rare transmission among humans, strains of *B. pseudomallei* in Australia have remained phylogenetically distinct from strains in Asia, Africa, and the Americas (*2*,*6*). Most reported instances of sequence type (ST) overlap between Asia and Australia are unrelated at the whole-genome level (*7*), with the exception of ST562 (*8*). Some STs in Southeast Asia occur over large geographic areas, such as along the Mekong and other rivers where erosion and washout from disturbed land might have contributed to *B. pseudomallei* dissemination (*2*,*9*,*10*).

Within Australia, most *B. pseudomallei* STs have a restricted geographic range (*11*). In the urban and rural areas of Darwin, STs have been found in the environment distributed across no more than 50 km (*12*). In Northern Australia, researchers have identified only 2 instances of long-range *B. pseudomallei* dispersal, spanning distances of 90 km and 460 km (*13*,*14*). *B. pseudomallei* isolates in the Northern Territory of Australia are very diverse, belonging to at least 379 reported STs (*12*). In this region, strains found in clinical and environmental samples exhibit similar levels of diversity (*11*). High species diversity of *B. pseudomallei* exists in urban Darwin; however, several STs, including ST109, ST36, ST132, and in recent years, ST553, have dominated among clinical and environmental isolates (*7*,*15*).

The Darwin Prospective Melioidosis Study has documented every culture-confirmed melioidosis case in the Top End region of the Northern Territory since 1989. In 2005, we reported the emergence of *B. pseudomallei* ST562 in urban Darwin (*8*). Genomic analyses revealed limited diversity among isolates and a very narrow geographic range, suggesting a single, recent introduction event from Asia (*8*). We describe the clinical manifestations and genomic epidemiology of *B. pseudomallei* ST562, which is now well-established in urban Darwin and causes a large proportion of melioidosis cases in the region.

## Methods

### Melioidosis Cases

We conducted this study at Royal Darwin Hospital, the referral center for the Top End region. The Top End is in the wet-dry tropics, ≈245,000 km^2^ in area, and sparsely populated. Darwin, the only city in the region, has a population of ≈122,000 persons; the remaining population lives in towns or remote communities separated by vast geographic distances. As part of the Darwin Prospective Melioidosis Study, we documented the demographic characteristics, risk factors, clinical features, and outcomes of 1,148 patients with culture-confirmed melioidosis during October 1, 1989–September 30, 2019 (*1*). We conducted multilocus sequence typing on isolates from 1,108 of 1,148 patients (https://pubmlst.org/organisms/burkholderia-pseudomallei) (*16*). Our study was approved by the Human Research Ethics Committee of the Northern Territory Department of Health and the Menzies School of Health Research, Darwin.

### *B. pseudomallei* Isolates

We analyzed *B. pseudomallei* ST562 sequences from 61 humans (including 3 with recurrent infection), 3 animals, and 4 environmental samples in the Top End; 5 isolates from humans in Hainan Province, China; and 1 isolate from a human in Pingtung County, Taiwan. We conducted whole-genome sequencing using HiSeq 2000, HiSeq 2500, HiSeq 3000, MiSeq, or NovaSeq 6000 (Illumina, Inc., https://www.illumina.com). We also analyzed the genome of a ST562 strain isolated from a water source in Haikou city, Hainan Province, in 1975 (*17*). In addition, we conducted a global phylogenetic analysis using 281 non-ST562 *B. pseudomallei* genomes available in public sources (Appendix 1 Table). We displayed the geographic distribution of Australia ST562 isolates using ArcGIS (https://www.arcgis.com/index.html) with shape files provided by the government of the Northern Territory.

### Statistical Analyses

We conducted all analyses using R version 3.6.0 (http://www.r-project.org). We used a 2-tailed Fisher exact test to conduct a bivariate analysis of demographic characteristics, underlying conditions, clinical features, and outcomes of 53 patients with ST562 and 387 patients with non-ST562 *B. pseudomallei* infection during October 1, 2004–September 30, 2019 in urban Darwin. We considered significant characteristics (i.e., p<0.05 in bivariate analysis) in a binomial multivariable generalized linear model with ST562 infection as the outcome. Because of the strong temporal structure to the data, we also included year of diagnosis as a continuous variable (Appendix 2).

### Bioinformatic Analyses

We conducted multiple sequence alignment and variant calling with Snippy version 4.3.6 (https://github.com/tseemann/snippy), using the closed ST562 MSHR5858 genome (*18*) (GenBank accession nos. CP008891–2) as the reference for ST562 phylogenetic analyses and the closed K96243 genome (*19*) (GenBank accession nos. BX571965–6) for the global analysis. We conducted maximum-likelihood phylogenetic analyses using IQ-TREE version 1.6.10 (*20*) and predicted regions of recombination using Gubbins version 2.3.4 (*21*). We used BEAST 2 (*22*) for temporal analysis of the core Australia ST562 alignment (Appendix 2).

## Results

### Australia *B. pseudomallei* ST562 Epidemiology

During 1989–2019, a total of 61 (5.5%) of 1,108 melioidosis cases were caused by *B. pseudomallei* ST562. After treatment completion, 3 (5%) patients had recurrent ST562 infection. Fifty-three (87%) ST562 patients resided in urban Darwin and 5 (8%) in an island community 81 km north of Darwin. In addition, 1 patient was evacuated from a remote community in East Arnhem Land, Northern Territory, Australia, 6 days after returning from a visit in Darwin; 1 patient lived in a rural community 37 km from Darwin; and 1 patient with an unknown travel history sought treatment at Katherine District Hospital (Katherine, Northern Territory, Australia), 317 km south of Darwin.

**Figure 1:**
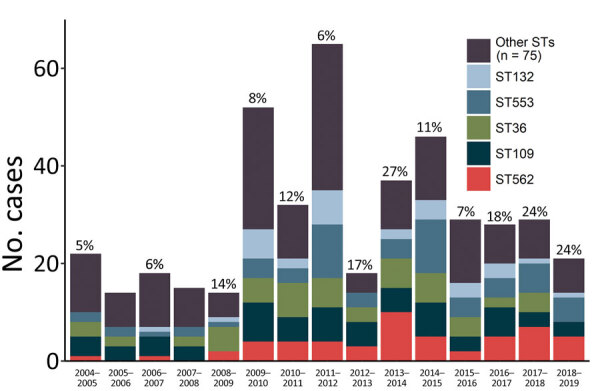
Distribution of melioidosis cases caused by various STs of *Burkholderia pseudomallei*, Darwin, Australia, 2004–2019. Twelve-month periods reflect the wet season then dry season and span October 1–September 30. ST, sequence type.

**Figure 2:**
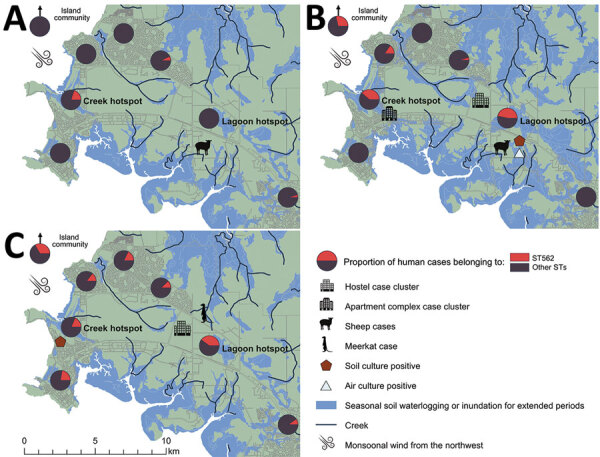
Proportion of melioidosis cases in humans caused by *Burkholderia pseudomallei* ST562, Darwin, Australia, 2004–2019. A) During October 2004–September 2009. B) During October 2009–September 2014. C) During October 2014–September 2019. ST, sequence type.

During 2005–2019, the proportion of human melioidosis cases caused by ST562 in urban Darwin gradually increased (Figure 1). These cases mostly occurred in 2 hotspot regions: suburbs surrounding a creek where 17 (30%) of 57 melioidosis cases were caused by ST562 and a lagoon where 11 (38%) of 29 cases were caused by ST562. The geographic distribution of cases changed over the 15-year period, moving initially from the creek hotspot to other regions in Darwin and to the island community (Figure 2). Records showed 2 case clusters with known epidemiologic links; the first cluster comprised 5 patients at a hostel in the lagoon hotspot during January 2014–March 2019 and the second comprised 2 persons from separate apartments in the same complex who were each found dead in their apartments on the same day in January 2014. *B. pseudomallei* ST562 was isolated from the autopsy samples of the 2 persons.

In addition, ST562 infections developed in 2 sheep at a veterinary facility in 2009 and 2014 (*8*) and in a meerkat at a wildlife park in 2015 (*23*). These cases occurred in the lagoon hotspot. Environmental sampling at these facilities did not reveal *B. pseudomallei* ST562. Furthermore, despite extensive systematic sampling across Darwin in 2017–2018, researchers found ST562 on only 2 occasions, both at the creek hotspot (*15*). During the investigation of a 2011 melioidosis case in a human, we isolated ST562 from air and soil samples from the lagoon hotspot (*24*). 

### ST562 Risk Factors and Clinical Features

Among 440 melioidosis patients in urban Darwin during October 1, 2004–September 30, 2019, a significantly higher proportion of patients with ST562 were Aboriginal or Torres Strait Islander (66% vs. 44%; p<0.01), lived in the suburbs surrounding the creek hotspot (33% vs. 11%; p<0.01) or the lagoon hotspot (21% vs. 5%; p<0.01), or reported hazardous alcohol consumption (59% vs. 40%; p = 0.02) (Table 1). In a generalized linear model that included these predictors, only residence in either of the 2 hotspot locations was a significant predictor of infection (Table 1). Pneumonia was the most common manifestation among patients with ST562 (76%) and non-ST562 infections (68%) (Table 2). Among male patients, 10 (32%) with ST562 had a prostate abscess, compared with 33 (15%; p = 0.02) men with non-ST562 infections. In total, 3 patients with ST562 infection died before hospitalization. The proportion of patients with bacteremia, septic shock, or death from melioidosis was not different among those with ST562 versus non-ST562 infection (Table 2).

**Table 1:** Demographic characteristics and risk factors for melioidosis caused by *Burkholderia pseudomallei* ST562, Darwin, Australia, October 1, 2004–September 30, 2019*

Characteristic	ST, no. (%)†			Bivariate model			Multivariable model	
	562, n = 53	Other, n = 387		OR (95% CI)	p value		OR (95% CI)	p value
Median age, y (range)	51 (13–85)	53 (1–97)		1.01 (0.99–1.02)	0.49			
Sex								
F	22 (42)	163 (42)		Referent				
M	31 (58)	224 (58)		0.98 (0.52–1.81)	>0.99			
Ethnicity								
Non-Indigenous persons	18 (34)	216 (56)		Referent				
Aboriginal or Torres Strait Islanders	35 (66)	171 (44)		2.45 (1.30−4.77)	<0.01		1.88 (0.94–3.77)	0.08
Hotspot‡								
Creek	17 (33)	40 (11)		3.83 (1.84–7.80)	<0.01		4.75 (2.22–10.19)	<0.01
Lagoon	11 (21)	18 (5)		5.02 (1.10–12.17)	<0.01		6.10 (2.39–15.54)	<0.01
Underlying condition								
Diabetes	28 (53)	177 (46)		1.33 (0.72–2.47)	0.38			
Hazardous alcohol consumption	31 (58)	156 (40)		2.08 (1.120–3.93)	0.02		1.72 (0.88–3.36)	0.11
Chronic lung disease	18 (34)	104 (27)		1.40 (0.71–2.67)	0.33			
Chronic kidney disease	9 (17)	53 (14)		1.29 (0.52–2.88)	0.53			
Congestive cardiac failure or rheumatic heart disease	3 (6)	34 (9)		0.62 (0.12–2.10)	0.60			
Malignancy	7 (13)	49 (13)		1.05 (0.39–2.52)	0.83			

*OR, odds ratio; ST, sequence type. †Values are no. (%), except as indicated. ‡Values missing for 1 patient with ST562 and 30 patients with other STs.

**Table 2:** Clinical features of melioidosis caused by *Burkholderia pseudomallei* ST562, Darwin, Australia, October 1, 2004–September 30, 2019*

Characteristic	ST, no. (%)			Bivariate	
	562, n = 53	Other, n = 387		OR (95% CI)	p value
Symptoms for <2 months	50 (94)	346 (89)		1.97 (0.59–10.32)	0.33
Localization					
Pulmonary	40 (75)	263 (68)		1.45 (0.73–3.06)	0.34
Abscess†					
Prostatic	10 (32)	33 (15)		2.73 (1.05–6.73)	0.02
Hepatic	1 (2)	13 (3)		0.55 (0.01–3.82)	>0.99
Splenic	2 (4)	25 (6)		0.57 (0.063–2.39)	0.76
Renal	3 (6)	9 (2)		2.51 (0.42–10.49)	0.17
Skin and/or soft tissue	3 (6)	51 (13)		0.40 (0.08–1.30)	0.17
Bone or joint	3 (6)	28 (7)		0.77 (0.14–2.64)	>0.99
Central nervous system	0	4 (1)		NA	>0.99
Severity					
Bacteremia¶	28 (55)	233 (61)		0.79 (0.42–1.49)	0.45
Septic shock	12 (23)	66 (17)		1.42 (0.64–2.94)	0.34
Death	7 (13)	35 (9)		1.53 (0.54–3.77)	0.32

*NA, not applicable; OR, odds ratio; ST, sequence type. †Missing data for 1 patient with non-ST562 *B. pseudomallei* infection. Prostate abscess data is for 31 men with ST562 and 223 men with other STs. ¶Missing data for 2 patients with ST562 and 3 patients with other STs.

### Australian *B. pseudomallei* ST562 Diversity

**Figure 3:**
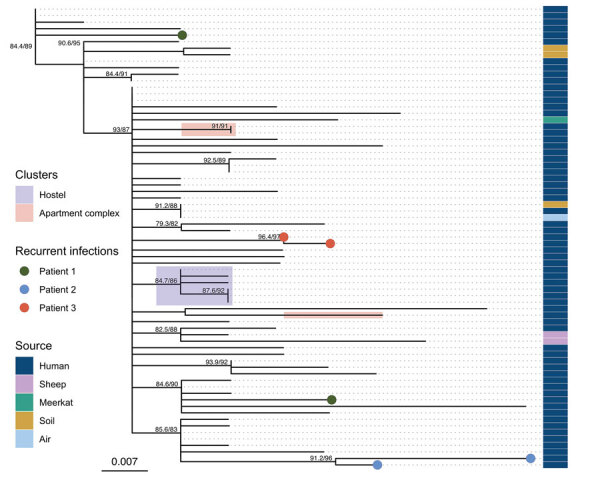
Maximum-likelihood phylogeny of *Burkholderia pseudomallei* sequence type 562 isolates from northern Australia, 2004–2019. Strain MSHR1967 (GenBank accession no. SRR2886997), the earliest sample, was used as the outgroup. Labels indicate nodes with approximate likelihood ratio >60 and ultrafast bootstrap >80. Scale bar indicates substitutions per site.

The 71 ST562 isolates from Australia were closely related with 141 single-nucleotide polymorphisms (SNPs) over a core alignment length of 7,071,987 nucleotides. These isolates were substantially less diverse than isolates of ST109, ST36, and ST132, which also are found in Darwin (*8*). The median pairwise difference among ST562 genomes was 5 SNPs (range 0–16 SNPs). We found a limited phylogenetic structure among the ST562 genomes, with multiple polytomies on maximum-likelihood analysis (Figure 3); we did not identify any recombination. The soil isolate collected from the lagoon hotspot, MSHR4681, differed by only 5 SNPs from the first soil isolate from the creek hotspot, MSHR10541, despite being collected 6 years and 12 km apart. Isolates from 3 patients (MSHR8799, MSHR9707, and MSHR11750) had no known epidemiologic links but were separated by 0 SNPs.

### *B. pseudomallei* ST562 Genomic Clusters

The ST562 isolates from cases associated with the hostel were phylogenetically clustered (Figure 3). The first 2 cases occurred 1 day apart in January 2014 and the third occurred in March 2014; isolates from these 3 cases differed by 0 SNPs. A fourth case in December 2014 differed from the first 3 isolates by 1 SNP and a fifth case in March 2019 differed by an additional SNP. This clade also included an ST562 isolate from a patient not initially known to have resided at the hostel; further investigation revealed that this patient had checked out of the hostel 6 days before being evacuated from a remote community for melioidosis treatment. Environmental sampling at the hostel did not detect ST562. We advised hostel staff regarding melioidosis prevention, including the importance of protective footwear and remaining indoors during storms.

The isolates from the 2 deceased persons from the apartment complex differed by 7 SNPs and did not fall within the same clade on the phylogenetic tree (Figure 3). However, an isolate from 1 of these patients was separated by 0 SNPs from a clinical isolate collected 10 months earlier from a patient who lived in the adjacent unit. Soil and water sampling of the apartment complex and its surroundings did not identify any *B. pseudomallei* ST562 isolates.

ST562 isolates from air and soil samples at a residence in the lagoon hotspot were separated by 0 SNPs. These isolates differed by 3 SNPs from a clinical isolate from a resident with bacteremic mediastinal melioidosis (*24*). Thirteen other clinical isolates were more closely related to the air and soil isolates than the clinical isolate from the resident; 1 isolate from a patient 8 years later was identical to the soil and air isolates. That patient lived 3.4 km downwind from the environmental sampling site.

### Recurrent *B. pseudomallei* ST562 Infections

Of the 3 recurrent ST562 cases, genomic analysis confirmed that 1 case was a new infection and 2 were relapses (Figure 3). The first patient was treated for melioidosis pneumonia in January 2009 and March 2016; isolates from these 2 episodes differed by 9 SNPs and belonged to different phylogenetic clades, suggesting that these illnesses were caused by independent infection events (*25*). The second patient had *B. pseudomallei* isolated from urine in December 2017. This patient was treated with intravenous therapy for 4 weeks with ceftazidime then meropenem, then for 12 weeks with oral doxycycline. In October 2018, *B. pseudomallei* was again isolated from this patient’s urine; the 2 isolates differed by 5 SNPs and belonged to the same clade, suggesting relapse. The third patient had acute pneumonia in January 2019 and was treated for 4 weeks with intravenous meropenem then ceftazidime, then for 12 weeks with oral trimethoprim/sulfamethoxazole. His symptoms improved and he returned to his community but was subsequently found dead in August 2019. His autopsy revealed pneumonia caused by an isolate differing from his original infection by only 1 SNP.

### *B. pseudomallei* ST562 Origin and Dispersal

**Figure 4:**
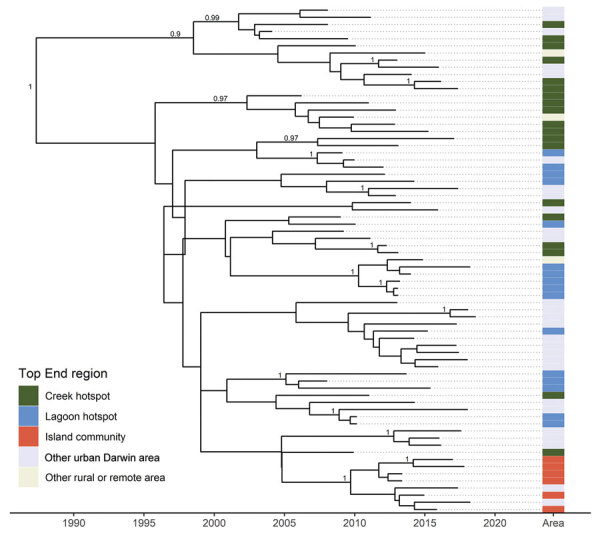
Maximum clade credibility tree of *Burkholderia pseudomallei* sequence type 562 isolates from northern Australia, 2004–2019. Labels indicate nodes with posterior support >0.8.

**Figure 5:**
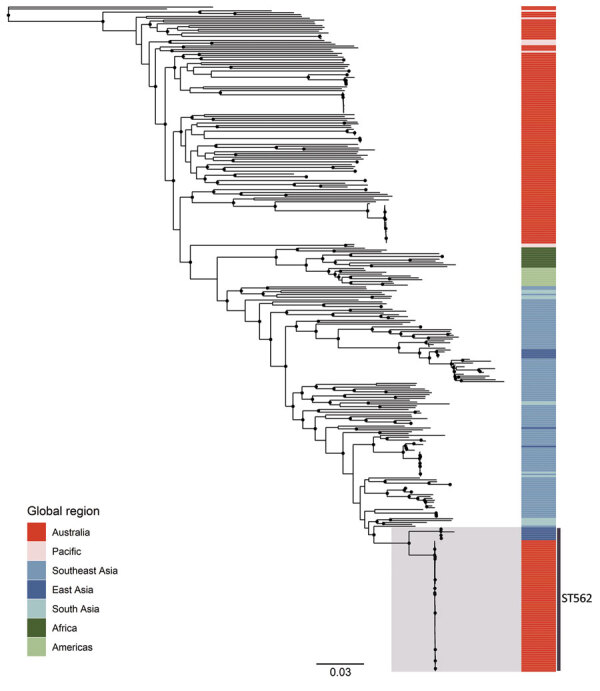
Maximum-likelihood global phylogeny of *Burkholderia pseudomallei* sequence type 562 isolates from northern Australia, 2004–2019, and genomes available in public sources (Appendix 1 Table). Strain MSHR5619 (GenBank accession no. ERR298346), which had the most divergent genome, was used as the outgroup. Black circles indicate nodes with approximate likelihood ratio >95 and ultrafast bootstrap >95. Colors indicate geographic origin of samples. Scale bar indicates substitutions per site. ST, sequence type.

The mean estimated clock rate for the 71 Australia *B. pseudomallei* ST562 isolates was 4.11 × 10^–8^ substitutions/site/year (95% highest posterior density [HPD] 2.0–6.2 × 10^–8^ substitutions/site/year) and the median estimate for the time to the most recent common ancestor was 1988 (95% HPD 1961–2001) (Figure 4). Isolates from the creek hotspot predominated on the deepest branching clades and were distributed throughout the phylogeny, indicating initial establishment in and dispersal from the creek hotspot. Isolates from patients from the island community formed a clade estimated to have diverged from a common ancestor in 2010 (95% HPD 2004–2014). The 5 isolates from Hainan and the isolate from Taiwan were not included in the molecular dating analysis due to poor clock signal; these isolates were distantly related to ST562 isolates from Australia, differing by 6,252–7,786 SNPs (964–1,453 SNPs when excluding recombinogenic regions). In the global *B. pseudomallei* analysis, *B. pseudomallei* ST562 isolates from Australia were most closely related to isolates from East Asia (Figure 5). The ST562 clade belonged to the larger Asian clade in the global phylogeny (*8*).

## Discussion

*B. pseudomallei* ST562 emerged in northern Australia in 2005, fifteen years after the Darwin Prospective Melioidosis Study began genomic surveillance (*1*,*8*). Initially, cases of ST562 in northern Australia mostly occurred in a creek hotspot before spreading across Darwin and to an island community to the north. A La Niña period of heavy rainfall during 2010–2012 was associated with increased melioidosis case numbers in Darwin (*26*). After this period, the geographic distribution and proportion of cases attributable to ST562 rose. Increased connectivity of waterways and wet conditions might have contributed to ST562 spread in Darwin during this time.

The clinical manifestations, symptom duration, and severity of melioidosis caused by ST562 were similar to those caused by non-ST562 infections, suggesting that host risk factors and route of acquisition contributed to clinical features more than differences in virulence profiles (*1*,*27*). The only difference in clinical manifestations was a larger proportion of male ST562 patients with prostate abscesses. Compared with the rate in Asia, the greater melioidosis survival rates observed in Australia are probably improved by greater access to treatment, including intensive care (*1*,*4*).

Genomic analysis of ST562 strains from Australia demonstrated very little diversity, suggesting a single introduction event with a probable origin in Asia (*8*). The only other characterized ST562 isolates in this study are from Hainan Province, China, and from Pingtung County, Taiwan. Comparative genomic analysis showed that the strains from China and Taiwan strains belonged the same clade but were distantly related to strains in Australia. Researchers have not identified any close relatives of ST562 strains in Australia; their precise origin within Asia remains uncertain.

We estimated that the most recent common ancestor of the ST562 strains in Australia, which indicates the possible time of introduction, occurred in 1988; however, the 95% HPD for this estimate was wide (1961–2001). The estimated clock rate of 4.11 × 10^–8^ substitutions/site/year was lower than previously reported. For example, previous reports estimated the rate for serial isolates in patients with cystic fibrosis as 4.9 × 10^–7^ substitutions/site/year (*28*) and for isolates from a 16-year chronic lung infection as 1.7 × 10^–7^ substitutions/site/year (*29*). For *B. pseudomallei* groups in Asia and the Americas, the estimated mutation rates range from 1.12 × 10^–6^ to 9.22 × 10^–7^ substitutions/site/year (*2*). The variation in these estimates might reflect the difficulty in identifying and excluding SNPs resulting from recombination, the different ecologic conditions and selective pressures of isolates, and inadequate sampling.

*B. pseudomallei* can exist in a viable but nonculturable state (*30*) and can persist in the environment in suboptimal conditions outside of regions to which it is endemic; the slow replication rate during these periods might contribute to its slow accumulation of mutations. Cases have sporadically occurred in temperate Western Australia, where 2 isolates from animals on different farms collected 17 years apart differed by just 1 SNP (*31*). In contrast, the bacteria can evolve rapidly during acute infection; in 1 patient, 8 SNPs and 5 small insertions/deletions developed in a 12-day period (*32*). We observed similar variability; for example, environmental and clinical samples collected 8 years apart differed by 0 SNPs, whereas isolates collected 10 months apart from the same patient differed by 5 SNPs. *B. pseudomallei* replication is probably greater in vivo, with the human host milieu placing the bacterium under greater selective pressure than the natural environment.

Previous epidemiologic investigations of melioidosis clusters in humans and animals suggest differences of <1 SNP from an implicated infecting source (*15*,*23*,*33*,*34*). In the investigation of 2 cases of infection with *B. pseudomallei* ST325 on the same rural property in northern Australian, there was 1 SNP difference between the 2 clinical isolates and the suspected environmental source, an unchlorinated water tank (*33*). In a fatal outbreak in a remote island community in northern Australia, 4 *B. pseudomallei* ST126 clinical isolates differed by <1 SNPs from an isolate from the town water supply (*34*). We confirmed case clusters at a hostel and an apartment complex by combining epidemiologic information and phylogenetic analysis, enabling the identification of previously unassociated cases. There was little diversity among *B. pseudomallei* ST562 isolates in northern Australia; many epidemiologically unrelated isolates differed by ≤1 SNP. Phylogenetic analysis was required for cluster identification.

Intercontinental dispersal of *B. pseudomallei* is an extremely unusual event, as demonstrated by the strong phylogeographic signal in the global phylogeny (*2*,*5*–*8*). The mode of ST562 transmission into northern Australia and then to an offshore island is unclear but could have been through soil, plants, animals, or humans (*8*) or through air during a severe weather event. The bacterium does not survive prolonged exposure to ultraviolet light, which probably limits aerial dispersal (*35*); many researchers consider long-range intercontinental spread through the air unlikely. However, *B. pseudomallei* has been isolated from air samples (*24*,*36*), suggesting that this route of transmission might be possible across relatively short distances. The dissemination of *B. pseudomallei* across islands in the Caribbean might have been mediated by hurricanes (*37*), and the dispersal of *B. pseudomallei* across northern Australia might be associated with tropical cyclones (*38*). Tropical cyclones occur every year in northern Australia, and melioidosis clusters can occur after these events (*31*,*39*). Clusters also have been associated with typhoons in Taiwan, where studies using multilocus sequence typing have suggested airborne dissemination from soil and an increase in human cases depending on the wind direction (*36*,*40*). The distance that *B. pseudomallei* can travel in such events remains uncertain.

Although *B. pseudomallei* colonization of humans is rare (*29*,*41*), the bacterium has been found in the feces of domestic and wild animals including wallabies, horses, and chickens (*42*,*43*), and in the beak of a healthy native peaceful dove (*Geopelia placida*) (*44*). A strong association exists between *B. pseudomallei* presence in soil and disturbance by horses, chickens, and pigs (*45*). *B. pseudomallei* has been imported into areas to which it is not endemic and that are associated with exotic animals (*46*,*47*), the most dramatic example of which was an outbreak in a zoo in Paris that caused the deaths of >2 humans and animals belonging to >10 different species (*43*,*48*). The outbreak spread from the Paris Zoological Park to the menagerie at the Paris Botanical Gardens and equestrian clubs across France. *B. pseudomallei* was isolated from horse manure in multiple gardens and from petri dishes placed near manure, suggesting aerosolization. Movement of horses for races contributed to the outbreak. 

Animal importation and migratory birds are possible modes by which ST562 could have arrived in Darwin. In the lagoon hotspot, 2 facilities that housed imported animals were the sites of 3 cases in animals of melioidosis caused by *B. pseudomallei* ST562. A horse racetrack and multiple equestrian clubs are also in the creek and lagoon hotspots. Both hotspots are habitats for water birds, many of which migrate to the region every year through Asia’s great flyways (*49*) and which could have carried *B. pseudomallei* ST562 to Darwin from Asia.

Although phylogenetic analysis confirms a single introduction event of Asian origin, how ST562 spread into northern Australia remains unknown. ST562 is now one of the most common *B. pseudomallei* STs in humans in urban Darwin. However, this ST rarely is isolated from the environment, including at sites associated with outbreaks. Further focused environmental sampling at key sites will help clarify ST562 epidemiology in northern Australia. The expanding capacity for genomic sequencing of *B. pseudomallei* will probably increase awareness of the ongoing global and regional dispersal of this bacterium and consequent melioidosis cases in humans and animals.

Appendix 1**.** *Burkholderia pseudomallei* sequences used in a study of melioidosis caused by sequence type 562 in Northern Australia.

Appendix 2 Additional methods used in study of *Burkholderia pseudomallei* sequence type 562, Northern Australia.
