# Supplementary material for: Emergence of Burkholderia pseudomallei Sequence Type 562, Northern Australia
Source: Emerg Infect Dis. 2021 Apr;27(4):1057–67. doi: 10.3201/eid2704.202716 (PMC8007296; doi:10.3201/eid2704.202716)
Supplement: Appendix 2 — Additional methods used in study of Burkholderia pseudomallei sequence type 562, Northern Australia. [file 20-2716-Techapp-s2.pdf]

Article DOI: https://doi.org/10.3201/eid2704.202716

Emergence of *Burkholderia pseudomallei* Sequence Type 562, Northern Australia

Appendix 2

Methods

Statistical Analyses

We used a bivariate analysis to show that 2 areas of Darwin, ethnicity, and history of hazardous alcohol consumption were associated with ST562 infection ($p<0.05$). We included these characteristics, along with year of diagnosis, in a binomial multivariable generalized linear model with ST562 infection as the outcome. We observed no evidence of collinearity. Using bootstrapped refitted residuals with the R package DHARMa (The R Project, https://cran.r-project.org), we found no evidence of overdispersion, outliers, or excessively influential observations. We tested a random intercept for the urban Darwin area in a multivariable generalized linear mixed model using the R package lme2 but did not improve the model fit and was not pursued further.

Bioinformatic Analyses

We used Snippy version 4.3.6 (https://github.com/tseemann/snippy) with thresholds for calling variants that included ≥10× coverage and ≥90% variant prevalence. We used IQ-TREE version 1.6.10 (*1*) to conduct a maximum-likelihood regression analysis using a generalized time-reversible model with 4 gamma categories; 1,000 ultrafast bootstrap approximation replicates; and 1,000 bootstrap approximate likelihood-ratio test replicates. We visualized and annotated trees using the R package ggtree (*2*).

We conducted temporal analysis on the core Australian ST562 alignment using BEAST 2 (*3*). We compared combinations of nucleotide substitution and clock models using nested sampling and calculation of Bayes factor. We selected a generalized time-reversible site model with 4 gamma categories, a relaxed clock with log-normal distribution of rates and a coalescent constant population model. We added constant sites to the .xml file using beast2_constsites

(https://github.com/andersgs/beast2_constsites). We undertook 10 replicates of the analysis, each with 800 million iterations. We sampled from the posterior every 80,000 iterations after an initial pre–burn-in of 8 million iterations. We combined logs using LogCombiner, with 15% burn-in, and resampled every 800,000th iteration. Effective sample sizes for all parameters were >200. We combined and resampled trees for a total of 8,500 trees, then used TreeAnnotator to calculate a maximum clade credibility tree. To test whether priors were driving results, we ran 10 analysis replicates with random tip dates with .xml files generated with the R package TipDatingBeast, and a replicate sampling from the prior with no sequence data; none of these analyses converged.

References

1. Nguyen LT, Schmidt HA, von Haeseler A, Minh BQ. IQ-TREE: a fast and effective stochastic algorithm for estimating maximum-likelihood phylogenies. Mol Biol Evol. 2015;32:268–74. PubMed https://doi.org/10.1093/molbev/msu300

2. Yu G, Lam TT, Zhu H, Guan Y. Two methods for mapping and visualizing associated data on phylogeny using ggtree. Mol Biol Evol. 2018;35:3041–3. PubMed https://doi.org/10.1093/molbev/msy194

3. Bouckaert R, Heled J, Kühnert D, Vaughan T, Wu CH, Xie D, et al. BEAST 2: a software platform for Bayesian evolutionary analysis. PLOS Comput Biol. 2014;10:e1003537. PubMed https://doi.org/10.1371/journal.pcbi.1003537
